# Strong CD4 T Cell Responses to Zika Virus Antigens in a Cohort of Dengue Virus Immune Mothers of Congenital Zika Virus Syndrome Infants

**DOI:** 10.3389/fimmu.2020.00185

**Published:** 2020-02-18

**Authors:** Catherine J. Reynolds, Patricia Watber, Camilla Natália Oliveira Santos, Danielle Rodrigues Ribeiro, Juliana Cardoso Alves, Adriana B. L. Fonseca, Ana J. B. Bispo, Roseane L. S. Porto, Kalliopi Bokea, Amélia Maria Ribeiro de Jesus, Roque Pacheco de Almeida, Rosemary J. Boyton, Daniel M. Altmann

**Affiliations:** ^1^Department of Infectious Disease, Faculty of Medicine, Imperial College London, London, United Kingdom; ^2^Molecular Biology Laboratory, Graduate Program in Health Science, University Hospital of the Federal University of Sergipe, Aracaju, Brazil; ^3^Microcephaly Clinic, Pediatric Division, University Hospital of the Federal University of Sergipe, Aracaju, Brazil; ^4^Molecular Biology Laboratory, Department of Medicine, University Hospital of the Federal University of Sergipe, Aracaju, Brazil; ^5^Department of Immunology and Inflammation, Faculty of Medicine, Imperial College London, London, United Kingdom

**Keywords:** zika virus, dengue virus, T cell epitope, flavivirus, cross-reactivity, microcephaly, adaptive immunity

## Abstract

**Background:** There is an urgent need to understand the complex relationship between cross-reactive anti-viral immunity, disease susceptibility, and severity in the face of differential exposure to related, circulating Flaviviruses. Co-exposure to Dengue virus and Zika virus in Brazil is a case in point. A devastating aspect of the 2015–2016 South American Zika outbreak was the dramatic increase in numbers of infants born with microcephaly to mothers exposed to Zika virus during pregnancy. It has been proposed that this is more likely to ensue from Zika infection in women lacking cross-protective Dengue immunity. In this case series we measure the prevalence of Dengue immunity in a cohort of mothers exposed to Zika virus during pregnancy in the 2015–2016 Zika outbreak that gave birth to an infant affected by microcephaly and explore their adaptive immunity to Zika virus.

**Results:** Fifty women from Sergipe, Brazil who gave birth to infants with microcephaly following Zika virus exposure during the 2015–16 outbreak were tested for serological evidence of Dengue exposure and IFNγ ELISpot spot forming cell (SFC) response to Zika virus. The majority (46/50) demonstrated Dengue immunity. IFNγ ELISpot responses to Zika virus antigens showed the following hierarchy: Env>NS1>NS3>C protein. Twenty T cell epitopes from Zika virus Env were identified. Responses to Zika virus antigens Env and NS1 were polyfunctional with cells making IFNγ, TNFα, IL-4, IL-13, and IL-10. In contrast, responses to NS5 only produced the immune regulatory TGFβ1 cytokine. There were SFC responses against Zika virus Env (1-20) and variant peptide sequences from West Nile virus, Dengue virus 1–4 and Yellow Fever virus.

**Conclusion:** Almost all the women in our study showed serological evidence of Dengue immunity, suggesting that microcephaly can occur in DENV immune mothers. T cell immunity to Zika virus showed a multifunctional response to the antigens Env and NS1 and immune regulatory responses to NS5 and C protein. Our data support an argument that different viral products may skew the antiviral response to a more pro or anti-inflammatory outcome, with an associated impact on immunopathogenesis.

## Introduction

There was considerable alarm caused by the Zika virus (ZIKV) epidemic which spread across more than 70 countries, especially the Americas, during 2015–2016. ZIKV is one of several viruses that are spread by *Aedes aegypti* mosquitoes in temperate climates (1). In many of the affected countries they can spread ZIKV, different serotypes of Dengue virus (DENV), and Yellow Fever virus (YFV) (2). There is a need to understand the complex relationship between human immunity and disease susceptibility in the face of differential exposure and immune memory to these viruses for which the patterns of immune cross-reactivity are as yet poorly mapped. Overlaid on these unknowns are the additional issues of *Aedes* population dynamics and the variable impact of climate, posing multi-faceted, and unresolved dilemmas (3). Areas of uncertainty include the cyclical patterns of cases for any given virus from rainy season to rainy season, raising concerns for example over when wide-scale ZIKV will return and what measures should be in place (3).

The phylogenetic relationships of the Flaviviruses are such that there is a degree of antigenic cross-reactivity due to sequence conservation across the family: ZIKV is closely related to DENV 1-4, and more distantly to WNV and YFV. This antigenic cross-reactivity increases the complexity of developing specific serodiagnostics, and has also stimulated considerable speculation with respect to implications for either protection or pathogenesis (4–7). In settings where individuals may have been exposed to symptomatic or asymptomatic infection by any of the 4 DENV serotypes, those facing subsequent exposure to ZIKV might be expected either to benefit from cross-reactive protection, to suffer enhanced disease (or enhanced disease to any fetus developing during the infection) through a mechanism such as antibody dependent enhancement (ADE), or neither of these. The impact of recognition of cross-reactive epitopes has been studied in the context of the response by B cells, CD4 and CD8 T cells. The consequences of cross-reactivity have been evaluated with *in vitro* neutralization studies and using *in vivo* models in mice or non-human primates. Findings differ somewhat depending on the specific DENV epitopes targeted by the human mAbs studied. In general, it appears that while some *in vitro* studies find evidence for ADE (8) with respect to DENV-immune, ZIKV infected patients, the effect is not detectable *in vivo* (9).

The question of prior Flavivirus exposure has especially profound implications with respect to the risk of delivering a child affected by congenital Zika virus syndrome in mothers infected by ZIKV during pregnancy. An initial report based on a murine model made the case that this risk, put as high as about 1 in 10, might correlate with lack of cross-reactive CD8 immunity from prior DENV infection (10). However, a later study proposed that, prior DENV antibodies might act to enhance ZIKV pathogenesis through placental damage leading to an increase in infected trophoblasts (11).

A number of observations have been reported with respect to the extent and consequences of CD4 and CD8 T cell epitope Flavivirus cross-reactivity (5, 12–17). In murine models, several CD8^+^ T cell epitopes from ZIKV were found to be cross-reactive with DENV and epitope responses stimulated by DENV2 were cross-reactive with ZIKV. This study suggested a CD8^+^ T cell-dependent mechanism, reducing viral titers in multiple tissues, responsible for cross-protection against ZIKV infection when there is prior DENV immunity (5). Gifroni and colleagues studied ZIKV epitope CD4 and CD8 responses in infected donors, with or without prior DENV immunological memory (13). They found that DENV immune donors showed ZIKV T cell responses that were larger, more rapid, and more cytotoxic. Another study of the T cell response to NS3 in sequentially DENV and ZIKV exposed people in West Africa similarly showed that prior infection increases the magnitude of response (14).

We and others have mapped the CD4^+^ T cell response to ZIKV, either in conventional, inbred mice, or in panels of HLA class II transgenic mice (15, 17). CD4 epitopes are found in all proteins. In particular, we found a response to an Env epitope that was shared across several different HLA class II restricting alleles. In several cases, ZIKV primed CD4 cells responded to the homologous epitope sequences from other viruses, including DENV1-4, WNV or YFV. Importantly, these cross-reactive responses could confer immune deviation, for example, the recall response of ZIKV-primed T cells by the Env DENV4 p1 epitope in HLA-DR1 transgenics resulted in a response skewed to IL-17A immunity. This suggested the possibility of a more complex relationship between sequential Flavivirus exposures, whereby responses may be pushed to become, not just different in terms of greater, or lesser magnitude, protection or pathogenicity, but of a differing effector phenotypic profile.

For the present study, we had the opportunity to study and map the ZIKV memory T cell responses (and cross-reactive epitopes from DENV) of a cohort of mothers from the Sergipe region of Brazil who had delivered affected children during the 2015–2016 ZIKV outbreak. This was an opportunity to study a clinical cohort of high specific interest; working with a cohort of symptomatically-infected mothers from the 2015 to 2016 ZIKV outbreak and living in an area of high DENV seroprevalence, we have regarded this observational study as one in the long and valuable clinical tradition of “uncontrolled case series,” rather than as a laboratory-controlled experimental design.

## Results

### Zika Virus Infected Cohort and Viral Serology

Characteristics of the clinical cohort of 50 individuals are shown in Table 1. All of the individuals in the cohort delivered an infant with microcephaly, the majority at full-term (37–52 weeks) of their pregnancy. For 15 individuals, the timing of onset of ZIKV infection was specified as during the 1st or 2nd trimester, one was infected during the 3rd trimester, and the remainder were unable to specify the exact timing of onset of ZIKV-related symptoms. The blood samples used in this study were collected at different time-points after delivery of the infant for each donor. The time interval between the birth of the infant and blood sample collection ranged from 1 to 24 months; for 25 out of 50 of the donors, the time between the birth of the infant and collection of the blood sample was up to 12 months.

**Table 1:** Cohort of mothers exposed to Zika virus during pregnancy in the 2015–2016 South American Zika outbreak that gave birth to an infant affected by microcephaly.

**Donor ID**	**Age (years)**	**City (Brazil)**	**Timing of ZIKV infection**	**ZIKV IgG test**	**DENV IgG test**
ARB 001-01	20–24	Itabaiana	Unknown	Positive	Positive
ARB 003-01	30–34	Itabaiana	Unknown	Positive	Positive
ARB 004-01	35–39	Itabaiana	Unknown	Positive	Positive
ARB 005-01	25–29	Itabaiana	First and second trimester	Positive	Positive
ARB 006-01	25–29	Santa Luzia do Itanhi	Unknown	Positive	Positive
ARB 007-01	20–24	Graccho Cardoso	First trimester	Positive	Positive
ARB 008-01	30–34	Itabaiana	Second trimester	Positive	Positive
ARB 009-01	25–29	Poço Redondo	Unknown	Positive	Positive
ARB 010-01	20–24	Nossa Senhora do Socorro	Unknown	Positive	Positive
ARB 012-01	30–34	Tobias Barreto	Second trimester	Positive	Positive
ARB 013-01	40–44	Itaporanga D'ajuda	Unknown	Positive	Positive
ARB 015-01	40–44	Umbaúba	First trimester	Positive	Positive
ARB 017-01	15–19	Nossa Senhora da Glória	Unknown	Positive	Positive
ARB 018-01	30–34	Tomar do Geru	Unknown	Positive	**Negative**
ARB 019-01	20–24	Itabaianinha	Unknown	Positive	Positive
ARB 021-01	25–29	Ribeirópolis	Unknown	Positive	Positive
ARB 025-01	15–19	Umbaúba	Unknown	Positive	Positive
ARB 026-01	20–24	Itabaiana	Unknown	Positive	Positive
ARB 031-01	15–19	Ponta dos Mangues	Unknown	Positive	Positive
ARB 032-01	30–34	Paripiranga	Unknown	Positive	Positive
ARB 033-01	25–29	Siriri	Unknown	Positive	Positive
ARB 035-01	35–39	Lagarto	Unknown	Positive	Positive
ARB 037-01	15–19	Lagarto	Unknown	Positive	Positive
ARB 038-01	25–29	Siriri	Eighth month	Positive	Positive
ARB 040-01	15–19	São Miguel do Aleixo	Unknown	Positive	Positive
ARB 041-01	25–29	Nossa Senhora do Socorro	Unknown	Positive	Positive
ARB 044-01	15–19	Pirambu	Unknown	Positive	Positive
ARB 046-01	20–24	Frei Paulo	Unknown	Positive	Positive
ARB 047-01	20–24	Itabaianinha	Unknown	Positive	Positive
ARB 048-01	15–19	Malhador	Unknown	Positive	Positive
ARB 049-01	30–34	Santa Luzia do Itanhi	First trimester	Positive	**Negative**
ARB 050-01	20–24	Poço Redondo	Unknown	Positive	**Negative**
ARB 051-01	15–19	Nossa Senhora do Socorro	Unknown	Positive	Positive
ARB 052-01	30–34	Aracaju	Fourth month	Positive	Positive
ARB 053-01	35–39	Riachão do Dantas	Third month	Positive	Positive
ARB 054-01	40–44	Simão Dias	Third trimester	Positive	**Negative**
ARB 055-01	20–24	Tobias Barreto	Third month	Positive	Positive
ARB 056-01	25–29	Nossa Senhora do Socorro	Second month	Positive	Positive
ARB 057-01	15–19	Pacatuba	Unknown	Positive	Positive
ARB 058-01	25–29	Aracaju	Unknown	Positive	Positive
ARB 059-01	15–19	Maruim	Unknown	Positive	Positive
ARB 060-01	–	–	Sixth month	Positive	Positive
ARB 061-01	30–34	Nossa Senhora da Glória	Sixth month	Positive	Positive
ARB 062-01	20–24	Nossa Senhora do Socorro	First month	Positive	Positive
ARB 063-01	20–24	Nossa Senhora do Socorro	Unknown	Positive	Positive
ARB 064-01	35–39	Nossa Senhora da Glória	Unknown	Positive	Positive
ARB 065-01	35–39	Nossa Senhora do Socorro	Unknown	Positive	Positive
ARB 067-01	25–29	Cristinápolis	Second month	Positive	Positive
ARB 068-01	15–19	Capela	Unknown	Positive	Positive
ARB 069-01	35–39	Itabaianinha	Unknown	Positive	Positive

Serum samples were tested using ZIKV-specific and DENV-specific IgG ELISAs. While there remain concerns about the genuine potential for serological cross-reactivity as a confounder in ELISA screening, these ELISAs have been standardized for use as recommended following trials by the Ministry of Health in Brazil. All the mothers in the cohort tested positive by ZIKV IgG ELISA. Of these, 4 tested negative by DENV-specific IgG by ELISA (Table 1). This commercial NS1 ELISA is validated for clinical use in Brazil under a working assumption that ZIKV IgG indicates ZIKV immunity and a result for DENV IgG, DENV immunity; however the authors acknowledge that there is uncertainty on this point in the field.

### Elispot Responses to ZIKV Antigens

**Figure 1:**
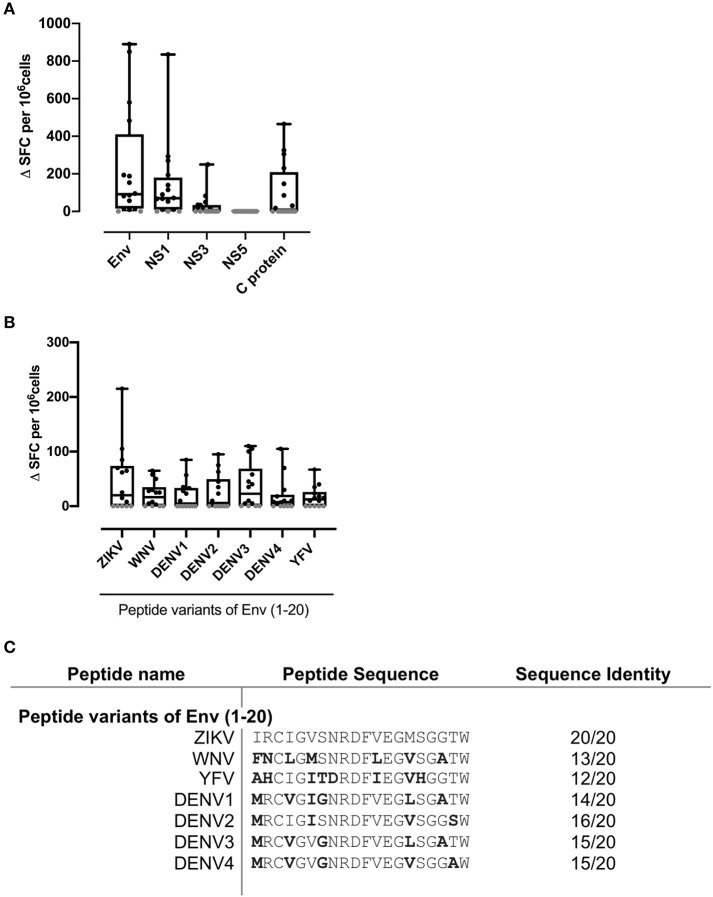
Strong IFNγ T cell recognition to ZIKV antigens Env, NS1, and flavivirus variants of Env (1-20). PBMC from 16 patients with a history of ZIKV infection were cultured with **(A)** the recombinant ZIKV antigens envelope protein (Env), non-structural proteins 1, 3, or 5 (NS1, NS3, or NS5), capsid (C) protein or **(B)** with ZIKV Env peptide 1 variants from the flaviruses ZIKV, West Nile virus (WNV), Dengue virus serotypes 1, 2, 3, or 4 (DENV1, DENV2, DENV3, or DENV4) or Yellow Fever virus (YFV). IFNγ responses were determined by ELISpot assay. An IFNγ response was defined as positive for a given individual if the delta spot forming cell (Δ SFC) value (that is, antigen response minus medium control) was >2SD of the mean of the no antigen control (black filled circle). Responses not defined as positive are shown as 0 (gray filled circle). **(C)** Amino acid sequence of Env 1-20 flavivirus variant peptides. Error bars indicate the mean and SD.

We initially tested 16 PBMC samples, selected at random from the donor pool and shown to have sufficient cell numbers and viability, and conducted IFNγ ELISpot assays to investigate responses to recombinant protein preparations of ZIKV Env, NS1, NS3, NS5, and C protein (Figure 1). Fifteen of 16 donors showed strong ZIKV antigen SFC responses. In terms of magnitude of response, the hierarchy of antigen recognition among responders was Env>NS1>NS3>C protein. Many donors displayed a T cell response to ZIKV ENV with mean SFC in excess of 200 SFC/10^6^. We did not detect an IFNγ ELISpot response to NS5 antigen.

### ELISpot Responses to Env (1-20) Epitope and Corresponding Variant Epitopes From Related Viruses

Our previous studies in murine models, either antigen-primed or ZIKV-infected, had highlighted an epitope within ZIKV Env (1-20), with amino acids capable of binding multiple HLAII alleles, six of the 7 common alleles tested, and recognized in the T cell response (17). The epitope within Env peptide 1–20 is one that overlies a protection-associated ZIKV CD8 epitope sometimes termed 294–302 (based on numbering of the full viral sequence (14). Furthermore, we showed in murine studies that ZIKV immune T cells could recognize the related Env (1-20) peptide sequence from several other Flaviviruses, but with the interesting caveat that this cross-reactive response could promote immune deviation, notably from IFNγ to IL-17 in response to DENV 3 and 4 variant peptides. We, therefore, tested responses of the same 16 donors to Env (1-20) and to the equivalent sequence variants from WNV, DENV1-4, and YFV (Figure 1). Nine out of 16 donors scored positive for Env (1-20) and the majority of individuals showed positive responses for the variant peptides. This analysis did not have the resolution to determine at the clonal level whether the DENV epitope responses constituted individual cells able to recognize both sequences cross-reactively, or distinct clones of ZIKV and DENV memory cells. All the individuals who showed a positive response to ZIKV and DENV1-4 peptide (1-20) were among those with evidence from serology of prior DENV immunity (Table 1: 007, 053, 044, 012, 026, 059, 060, 040, and 031). Individuals who responded to ZIKV Env (1-20), but not the variant DENV epitopes were donors 010 and 018. It can be seen from Table 1 that donor 010 was DENV seropositive, while donor 018 was without serological evidence for prior DENV exposure. These findings are compatible with the notion of SFC responses to the DENV1-4 variant epitopes as representing either DENV1-4 memory or cross-reactive priming by ZIKV. It would have been desirable to confirm T cell responses using a complementary approach such as intracellular cytokine staining (ICS) flow cytometry, but with ethical approval allowing one vial of <20 × 10^6^ cells from each donor, this was not possible.

### Differential Cytokine Profiles in ZIKV Antigen Responses

**Figure 2:**
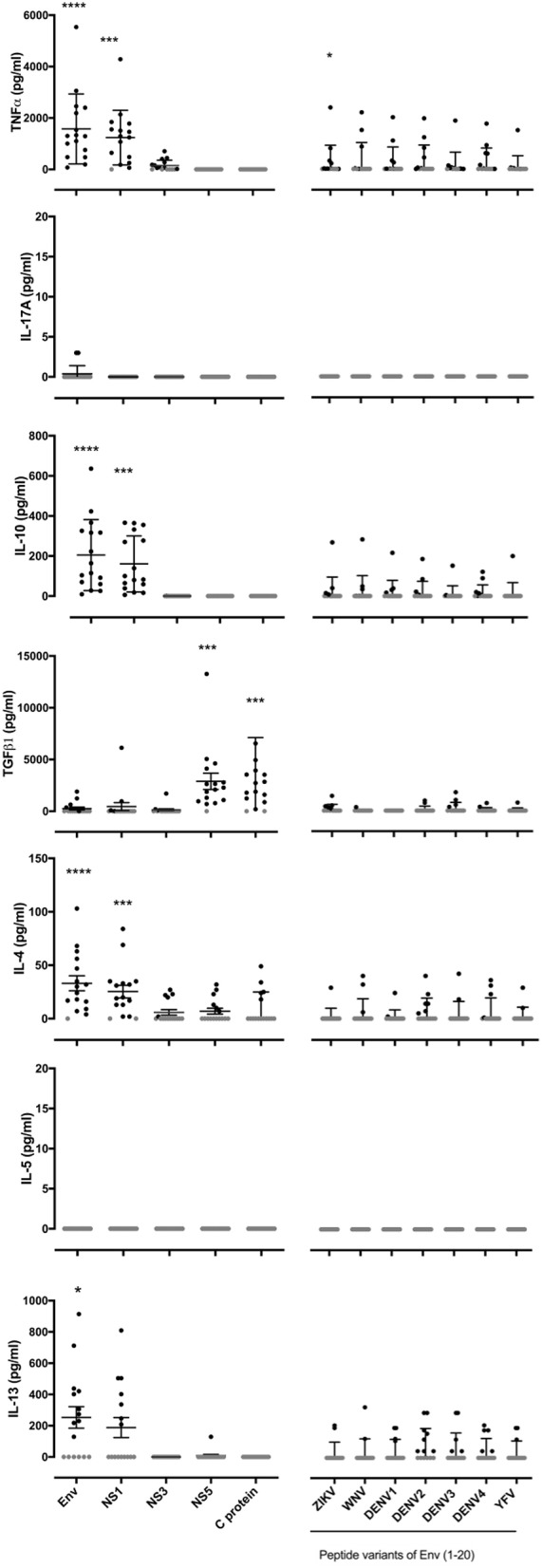
Polyfunctional cytokine responses to ZIKV antigens Env and NS1 while NS5 and C protein antigen specific responses limited to TGFβ. PBMC from 16 patients with a history of ZIKV infection were cultured with the recombinant ZIKV antigens envelope protein (Env), non-structural proteins 1, 3 or 5 (NS1, NS3, or NS5), capsid (C) protein or with ZIKV Env (1-20) variants from the flaviruses ZIKV,West Nile virus (WNV), Dengue virus serotypes 1, 2, 3, or 4 (DENV1, DENV2, DENV3, or DENV4) or Yellow Fever virus (YFV). After 24 h of culture, cell culture supernatants were collected and levels of TNFα, IL-17A, IL-10, TGFβ, IL-4, IL-5, and IL-13 measured by Luminex® assay. Data are presented minus the cytokine concentrations measured for the no antigen control samples for each individual. Responses that were zero are indicated using a gray filled circle. Statistical significance between negative control samples and protein antigen or peptide stimulation was determined using a Wilcoxon matched-pairs signed rank test. *p < 0.05, **p < 0.005, ***p < 0.0005, ****p < 0.0001.

We next investigated the cytokine profiles of responses elicited by this panel of ZIKV antigens and Env (1-20) peptide and its variants (Figure 2). Our earlier data in Env primed or ZIKV infected mice had shown that specific epitopes, and especially, recall with variant epitopes from related viruses, could be associated with divergent cytokine profiles (17). None of the human donor T cell responses studied here produced detectable IL-17A or IL-5. Responses to recombinant Env and NS1 antigens in virtually all individuals were noteworthy for high TNFα, IL-10, IL-4, and IL-13, in addition to IFNγ as already demonstrated by ELISpot analysis. While it was not possible to repeat the analysis at the single-cell level by flow, the findings are best explained by a highly polyfunctional T cell response to ZIKV.

Interestingly, we identified highly differential cytokine responses between different viral antigens: the majority of individual donors showed a strong TGFβ response elicited by NS5 and C protein, but not by the other antigens tested. Responses to NS5 had been silent with respect to IFNγ ELISpot. In several individuals, the response elicited by the DENV 1-4 variants of Env (1-20) involved TGFβ, IL-10, IL-4, and/or IL-13, compatible with a component of the response that is mediated by Treg and Th2 subsets. We note that the vast majority of donors were seropositive in the DENV-specific ELISA, so that we are unable by this assay to distinguish between recall responses through DENV T cell memory, and cross-reactive responses of ZIKV-specific T cells.

### T Cell Responses to Specific ZIKV Peptide Epitopes

**Figure 3:**
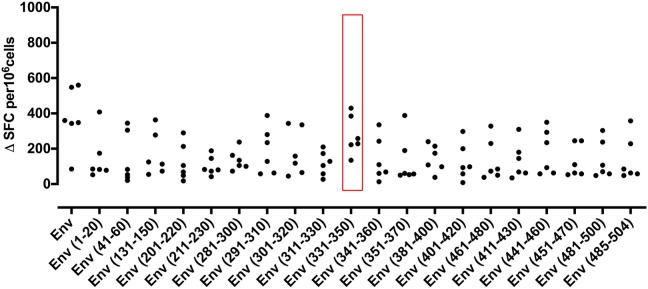
IFNγ T cell responses to twenty peptides identify frequent epitopes from ZIKV Envelope protein. PBMC from 6 patients with a history of ZIKV infection were cultured with individual 20 mer peptides from ZIKV Envelope protein (Env). IFNγ responses were determined by ELISpot assay. An IFNγ response was defined as positive for a given individual if the delta spot forming cell (Δ SFC) value was >2SD of the mean of the no antigen control. Responses not defined as positive are shown as 0.

**Figure 4:**
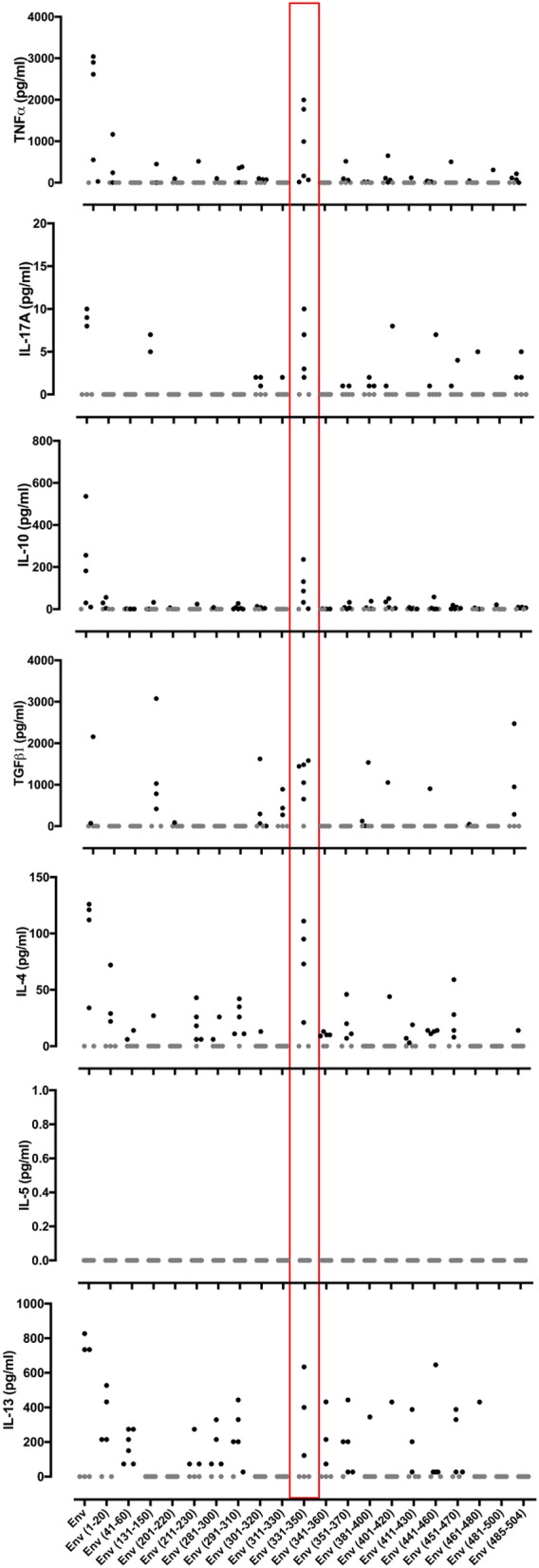
Cytokine responses to different peptides from the ZIKV Envelope protein. PBMCs from 6 patients with a history of ZIKV infection were cultured with individual 20 mer peptides from ZIKV Envelope protein (Env). After 24 h of culture, cell culture supernatants were collected and levels of TNFα, IL-17A, IL-10, TGFβ, IL-4, IL-5, and IL-13 measured by Luminex® assay. Data are presented minus the cytokine concentrations measured for the no antigen control samples for each individual. Responses that were zero are indicated using a gray filled circle.

In an effort to define individual T cell epitopes accounting for the response to ZIKV Env in the majority of infected individuals screened, we used samples from additional donors to map responses at the level of individual peptides. As the full overlapping peptide panel for this sequence contains 50 peptides and the PBMC sample cell numbers were limiting, we opted to screen donor ELISpot responses to a subset of the 20 peptides. To select which peptides to analyse we ranked the peptides using data derived from NetMHCIIpan 3.2 (www.cbs.dtu.dk/services/NetMHCIIpan) to predict the relative strength of CD4 binding for epitopes within ZIKV Env for the five most common MHC class II alleles in Sergipe Brazil (DRB1^*^13, ^*^07, ^*^11, ^*^04, 15 as identified using AlleleFrequencies.net) and HLA-DRB1^*^01. We also included data from our published relative peptide binding data (17) and epitopes published by others (18). All twenty of the peptides tested were confirmed as containing a T cell epitope recognized by several donors (Figure 3). The most prominent epitope in terms of percent responders and frequency of spot forming cells was Env 331-350. All six individuals tested responded to this peptide. We next investigated the extent to which the detected responses to Env peptides encompassed release of other cytokines (Figure 4). The response to Env 331-350 involves a polyfunctional release of TNFa, IL-17A, IL-10, TGFb, IL-4, and IL-13. In some donors the responses to epitopes Env 211-230, Env 281-300, Env 291-310, and Env 451-470 includes IL-4 and IL-13 while the response to epitope Env 131-150 involves TGFb, and Env 41-60 includes IL-13. These results indicate highly differential polyfunctional responses to different Env peptide epitopes.

## Discussion

In the present study we had the opportunity to analyze T cell responses to ZIKV antigens in a cohort of mothers who had suffered demonstrable infection during the recent ZIKV outbreak and delivered babies affected by congenital Zika virus syndrome. We currently lack additional, local, comparator groups needed to analyse implications of these immune responses comprehensively. For this reason, we regard the very real value of this study as being in the nature of an observational, “case series study” (19), rather than as a conventional, controlled study. One would ideally further analyse how these patterns of response differ from those in infected mothers who gave birth to unaffected babies and how might responses compare between definitively stratified groups for presence or absence of prior DENV infection? The latter comparison addresses an important mechanistic question generated by murine model studies of laboratory infection, but is extremely difficult to address in the “real-life” setting of this part of Brazil, where the majority of people may be unaware of asymptomatic DENV exposure.

Brazil is one of many countries in which Aedes mosquitoes can spread several Arboviruses, notably in this context, the phylogenetically and antigenically related species, ZIKV and DENV1-4. When the potential for contemporaneous spread of these different viruses is combined with the facts that many exposed individuals may suffer asymptomatic disease, be unaware of their exposure, and that, despite strenuous efforts, unequivocally differential clinical serodiagnostics remain a challenge, unraveling the interactions between different components of virus-specific immunity is not trivial. At its simplest, the question of interaction between DENV and ZIKV immunity may be framed as one of whether the immune repertoires are additive and cross-reactively protective, pathogenic through a variant of ADE, or poorly cross-reactive and thus independent of each other. Conflicting datasets in this regard have emerged from *in vitro* and *in vivo* studies, including mouse and non-human primate challenge models. A specific context for consideration of this matter has been evaluation of why it is that a minority of mothers who are infected with ZIKV during pregnancy go on to deliver a child which is affected by congenital ZIKV syndrome, especially microcephaly. A hypothesis that has been put forward is that the affected births are more likely among those mothers with no prior cross-reactive immunity through DENV exposure (10). Almost all of the mothers in our cohort who delivered affected babies were seropositive for both ZIKV and DENV. We are, therefore, confident in our description of this cohort as one containing a majority of individuals with prior immunity to DENV. However, recent analysis of a Brazilian cohort of congenital Zika virus syndrome mothers reported reduced DENV seroprevalence with a lower number of neutralizing serotypes (20). While the overwhelming likelihood in our Sergipe cohort is that DENV exposure preceded ZIKV exposure and did not occur in the months between the birth and donation of the blood sample, we cannot formally exclude the possibility that DENV exposure could have taken place after the ZIKV illness and that may have skewed or altered the responses in some way.

We acknowledge that it cannot be proved unequivocally that affected births were due to ZIKV exposure, not least since many exposures were asymptomatic. The 2015–2015 ZIKV outbreak coincided with an unprecedented rise in microcephaly births, these occurring in others who had shown evidence of ZIKV infection during the pregnancy. However, in only a tiny minority of cases was it possible to take steps to investigate a causal relationship. All of the mothers in this cohort were seen through the pediatric Service at the University Hospital of the Federal University of Sergipe, followed up through the Congenital Zika Syndrome unit. This follow-up included full neurological assessment, cephalic perimeter, exclusion of other sources of neurological injury, and exclusion of infection by CMV, Toxoplasma, Rubella or HSV.

Although we had limited numbers of PBMC from each donor, we have sought to take some of the first steps to addressing questions about T cell immunity in symptomatic ZIKV infection. We have been able to look at the hierarchy of T cell recognition of viral antigens, the range of cytokine responses to these antigens, the epitopes within Env and, to a limited extent, the relationship to prior immunity to DENV1-4 serotypes. While many attempts to examine CD4 T cells responses in ZIKV-exposed donors have relied on screening large peptide pools, we here adopted the strategy of analyzing responses to individual recombinant protein antigens, and individual peptides. Koblischke and colleagues previously mapped responses to a C/PrM/E peptide pool using PBMC from 14 ZIKV^+^ travelers returning to Europe following recovery from acute infection (21). This showed a diverse spread of epitope recognition, with some degree of focused recognition in the region of C protein amino acids 77–107. Analysis in an infected cohort from Senegal focused on analysing responses to NS3, showing high frequency ELISpot responses, including a component targeted to the NS3 helicase region and cross-reactive between DENV and ZIKV-immune donors (16).

The hierarchy of T cell antigen recognition observed in our study is different from previous published reports. Grifoni et al. showed that most of the NS responses in ZIKV exposed, DENV naïve subjects were directed against NS2a. In ZIKV/DENV exposed subjects, responses were found throughout the NS proteins, in roughly equal numbers (13). Earlier work, based on use of peptide pools, had found T cell recognition of ZIKV NS5 epitopes. We found a hierarchy of T antigen recognition whereby polyfunctional Env and NS1 recognition dominated. T cell recognition was associated with strong IFNγ, TNFα, IL-4, IL-13, and IL-10 responses. We could find no recognition of NS5 by IFNγ ELISpot. Rather, NS5 T cell recognition in our patient cohort was associated with a strong TGFβ1 response to NS5 and C protein. We did not have additional vials of cells to check the cellular origin of the TGFβ1 release by ICS, but assume that this profile may indicate preferential activation of ZIKV-specific Tregs by NS5 and C protein. Whether Treg activation is protective or detrimental in this context will require more detailed dissection of the underlying immunopathogenesis. As with DENV infections, the case can be made in ZIKV infections that the more severely symptomatic cases may be those in which there is a more exuberant, poorly regulated response (22–24). We cannot offer a definitive explanation for the observed differences between antigen hierarchies in the various published studies, but this may well be attributable that most previous work was reliant on decoding mixed peptide pools, whereas our study started from recombinant protein antigens.

The epitope mapping of individual peptides from ZIKV Env confirmed some previously identified epitopes as well as identifying several new ones. The epitope defined within Env (1-20) is noteworthy, since it overlaps a previously defined MHCI-restricted epitope previously described by us stimulating both CD4 and CD8 responses in HLA transgenic lines and shown to bind a number of different HLAII heterodimers. The epitope is relatively conserved within the Env sequence of several other Flaviruses, including DENV1-4. Our analysis of T cell responsiveness to ZIKV Env (1-20) and the variant sequences from DENV serves to underline the complexity of the immunological relationship between memory to these related sequences: while there has been previous conjecture as to the extent to which responses may be either additive in conferring protection, or pathogenic in terms of the potential to support ADE, one must also add the confounder of responses that become skewed to an alternate, regulatory programme.

Our initial epitope mapping of the T cell response to ZIKV Env identified epitopes within 20 different peptides, each recognized by several different donors. ZIKV Env can thus be regarded a relatively T cell epitope-rich antigen. Virtually all ZIKV immune donors whose T cell responses were analyzed at the level of responses to individual peptides showed an extremely strong response to Env (331-350). This common, high-frequency response was especially noteworthy for its breadth of polyfunctionality, showing induction of strong cytokine release with respect to IFNγ, TNFα, IL-17A, IL-10, IL-4, IL-13, and TGFβ. Polyfunctionality of response is generally considered a correlate of protection in immunity and has previously been highlighted as a feature of ZIKV-specific T cells (25).

In summary, this study shows that a cohort of ZIKV-infected mothers who delivered infants with microcephaly during the 2015–2016 outbreak in Brazil comprised a group who showed evidence of what we presume to be prior DENV immunity, suggesting that microcephaly can occur in DENV immune mothers. We found a hierarchy of T cell recognition to recombinant ZIKV antigens in the order Env>NS1>NS3>C protein in terms of IFNγ responses and NS5, C protein in terms of TGFβ responses. It is noteworthy that some antigen responses were associated with an alternate cytokine profile, such as the more regulatory signal elicited by NS5 and C protein. Thus, different viral products may arguably skew the antiviral response to a more pro- or anti-inflammatory outcome, with associated impact on immunopathogenesis.

## Materials and Methods

### Study Design

During 2017, peripheral blood samples were collected at the University Hospital of the Federal University of Sergipe, Brazil, from a cohort of 50 females who had given birth to a child affected by Congenital ZIKV syndrome, specifically, microcephaly. Peripheral blood mononuclear cells (PBMC) were isolated by Ficoll-Paque density gradient centrifugation and frozen for later use. Serum was collected for DENV and ZIKV ELISAs at the University of Sergipe labs. Frozen cell vials were then shipped to the Imperial College London for ELISpot and cytokine assays.

### Ethics Statement

This study was carried out in accordance with the recommendations of the Declaration of Helsinki. All blood donors gave written, informed consent. The study was approved by the Ethics and Research Committee of the Federal University of Sergipe (CAAE 54835916.2.0000.5546) and all patients provided informed consent for their participation in the study.

### Clinical Cohort

Fifty female donors who had given birth to infants with microcephaly were recruited with full written, informed consent from the towns around the University Hospital of the Federal University of Sergipe. A clinical history was taken, including the date of onset of symptomatic ZIKV infection and infants' diagnosis. The maternal blood samples were collected at between 1 and 24 months after delivery of the infant affected by congenital Zika syndrome.

### Cohort Serology

ELISAs were carried out to measure DENV IgG and ZIKV IgG (Euroimmun, Medizinische Labordiagnostika AG, Germany) (25, 26). For serological assays we used Anti-Zika Virus ELISA (IgG) and an Anti-Dengue Virus ELISA (IgG) and followed the manufacturer's instructions. These assays are validated for clinical use as non-cross-reactive insofar as they are the recommended test adopted by the Ministry of Health in Brazil. In brief, the samples were diluted 1:100 in sample buffer and incubated at 37°C (Anti-Zika Virus assay) or at room temperature (Anti-Dengue Virus assay) for 1 h. The optical density was measured at 450 and 630 nm using an Epoch spectrophotometer (BioTek®). Results were analyzed semi-quantitatively by calculating a ratio of the extinction value of the control or patient sample over the extinction value of the calibrator. The samples were categorized as negative (ratio < 0.8), borderline (ratio ≥ 0.8–< 1.1) or positive (ratio ≥ 1.1).

### Recombinant Antigens and Peptide Panels

Sequences of the envelope protein (Env), capsid protein (C), and non-structural proteins NS1, NS3, and NS5 of ZIKV were taken from the Brazilian isolate, GenBank accession no. AMH87239.1. After codon optimisation, recombinant proteins were expressed in *Escherichia coli* and purified by His-Tag. The hydrophobic transmembrane domain (456–504) of Env was not included to improve protein expression, solubility and stability. (Biomatik, Cambridge, ON, Canada) (17). While generation of recombinant proteins in E. coli may arguably impact on correct folding and glycosylation, the assumption in the field is that any impact of this on the ability to process antigen and generate T cell epitopes is marginal. Synthetic peptides, 20 amino acids (aa) in length and overlapping by 10 aa were generated for each of the recombinant proteins (GL Biochem, Shanghai, China) (17). Flavivirus variants of Env (1-20) for WNV (accession no. AFJ05105.1), YFV (accession no. AIZ07887.1), DENV 1 (accession no. AKQ00039.1), DENV 2 (accession no. AKQ00040.1), DENV 3 (accession no. ACO06174.1), and DENV 4 (accession no. AKQ00037.1) were also synthesized (17).

### ELISpot Analysis

T cell responses to antigen were inferred by IFNγ ELISpot spot forming cells (Mabtech, Sweden). One hundred and eighty thousand PBMC per well were assayed in duplicate or triplicate using pre-coated 96-well polyvinylidene difluoride (PVDF) plates. Protein or peptide were added to a final concentration of 25 μg/ml and plates were cultured at 37°C and 5% CO_2_ for 24 h. Cells plus cell culture media alone were added to negative control wells; anti-CD3 to a final concentration of 1 μgml was added as a positive control for cell viability and responsiveness. Prior to assay development, supernatants were collected from the cell culture plates and stored at −40°C for additional cytokine analysis. Following plate washing, IFNγ secretion was detected by incubation with an HRP conjugated antihuman-IFNγ detection antibody at room temperature for 2 h. Spots were revealed using a BCIP/NBP-plus substrate solution. IFNγ spots were quantified using an AID ELISpot reader (Autoimmun Diagnostika GMBH, Germany). Results were calculated as spot forming cells (SFC)/million PBMC after subtraction of the number of spot forming cells observed following culture with cell culture media alone (that is, ΔSFC). Responses were defined as positive if SFC/million PBMC was >2SD of the mean of the negative control wells for each individual tested. We were not able to analyse samples from all 50 donors side-by-side in every given set of assays; due to low cell numbers in many samples, we, therefore, analyzed randomly selected vials in different parts of the study, for example antigen responses vs. Env peptide mapping.

### Cytokine Analysis

Supernatants from PBMC cultured with individual proteins or peptides were collected prior to ELISpot assay development and levels of TNFα, IL-17A, IL-10, IL-4, IL-5, IL-13, and TGFβ were measured using Luminex® assay kits (Bio-Techne, USA) on a Bio-Plex 200 instrument (Bio-Rad Laboratories, Ltd, UK). Cytokine concentrations in response to protein or peptide were calculated by subtracting values obtained for negative control cultures. IFNγ was not assayed in supernatants as findings would have been confounded by consumption of IFNγ in prior ELISpot assays.

### Statistical Analysis

For cytokine data, statistical significance between negative control samples and protein antigen or peptide stimulation was determined using a Wilcoxon matched-pairs signed rank test and Graphpad Prism 7 software.

## Data Availability Statement

All datasets generated for this study are included in the article/supplementary material.

## Ethics Statement

The studies involving human participants were reviewed and approved by Ethics and Research Committee of the Federal University of Sergipe (CAAE 54835916.2.0000.5546). The patients/participants provided their written informed consent to participate in this study.

## Author Contributions

CR and PW developed and performed experiments, analyzed and interpreted data, and helped prepare the manuscript. KB helped prepare the introduction to the manuscript. AF, AB, and RP recruited the clinical cohort for the study. CS, DR, JA, AJ, and RA clinically characterized patients for the study including DENV IgG and ZIKV IgG ELISA. RA designed the study, clinically characterized the patient cohort, interpreted the data and helped prepare the manuscript. RB and DA conceived and designed the study, interpreted the data, and wrote the manuscript. RB and DA supervised the research and contributed equally to the study. All the authors discussed the results and commented on the manuscript.

### Conflict of Interest

The authors declare that the research was conducted in the absence of any commercial or financial relationships that could be construed as a potential conflict of interest.
